# Effect of temperature and water activity on the production of fumonisins by *Aspergillus niger *and different *Fusarium *species

**DOI:** 10.1186/1471-2180-9-281

**Published:** 2009-12-31

**Authors:** Jesper M Mogensen, Kristian F Nielsen, Robert A Samson, Jens C Frisvad, Ulf Thrane

**Affiliations:** 1 Center for Microbial Biotechnology, Department of Systems Biology, Technical University of Denmark, Søltofts Plads 221, DK-2800 Kgs. Lyngby, Denmark; 2 CBS-KNAW Fungal Biodiversity Centre, Uppsalalaan 8, NL-3584 CT Utrecht, The Netherlands

## Abstract

**Background:**

Fumonisins are economically important mycotoxins which until recently were considered to originate from only a few *Fusarium *species. However recently a putative fumonisin gene cluster was discovered in two different *Aspergillus niger *strains followed by detection of an actual fumonisin B_2 _(FB_2_) production in four strains of this biotechnologically important workhorse.

**Results:**

In the present study, a screening of 5 *A. niger *strains and 25 assumed fumonisin producing *Fusarium *strains from 6 species, showed that all 5 *A. niger *strains produced FB_2 _and 23 of 25 *Fusarium *produced fumonisin B_1 _and other isoforms (fumonisin B_2 _and B_3_). Five *A. niger *and five *Fusarium *spp. were incubated at six different temperatures from 15-42°C on Czapek Yeast Agar +5% salt or Potato Dextrose Agar. *A. niger *had the highest production of FB_2 _at 25-30°C whereas *Fusarium *spp. had the maximal production of FB_1 _and FB_2 _at 20-25°C. Addition of 2.5-5% NaCl, or 10-20% sucrose increased the FB_2 _production of *A. niger*, whereas addition of glycerol reduced FB_2 _production. All three water activity lowering solutes reduced the fumonisin production of the *Fusarium *species.

**Conclusion:**

The present study shows that the regulation of fumonisin production is very different in *A. niger *and *Fusarium*, and that food and feeds preserved by addition of sugar or salts may be good substrates for fumonisin B_2 _production by *A. niger*.

## Background

The fumonisins were discovered in 1988 and are divided in four series A, B, C, P [1-3]with the B_1 _(FB_1_), B_2 _(FB_2_) and B_3 _(FB_3_) as the most abundant naturally occurring homologues [4,5]. They were first isolated from *Fusarium verticillioides *(= *F. moniliforme *pro parte [6]) strain MRC 826 by Gelderblom *et al*. [7]. FB_1 _is mainly produced by *F. verticillioides *and *F. proliferatum *[8]. However, production of type B fumonisins by other *Fusarium *spp. has also been reported, e.g. from *F. dlaminii*, *F. napiforme*, *F. nygamai *and *F. oxysporum *[8-10]. Fumonisins are important mycotoxins because they are suspected to cause human and animal toxicoses by the consumption of contaminated corn-based food and feeds [11]. Fumonisins have been shown to induce outbreaks of equine leukoencephalomalacia in horses and pulmonary edema and hydrothorax in pigs [5,12]. The fumonisins are structurally similar to sphingolipids and have shown to inhibit the sphingolipid biosynthesis via the ceramide synthase pathway [13,14]. To avoid possible health risks, the U.S. Food and Drug Administration recommends that corn products should not be used for human consumption when contaminated with more than 2-4 mg/kg total fumonisins (depending on the product) [15]. whereas EEC has a regulatory limit of 0.2-2 mg/kg (depending on the product) [16].

Fumonisins produced by *Fusarium *species have been isolated from corn [1] and corn based products [11] such as tortillas [17] and beer [18], as well as other commodities like rice [19], black tea leaves [20], asparagus [21] and pine nuts [22].

Factors that affect the production of fumonisins in *Fusarium *have been well studied, and include solid substrates [23], liquid substrates [24], temperature [25-27], water activity (a_w_) [27,28], pH [29], addition of nitrogen repressor [30], aeration of the substrate [29] and addition of fumonisin precursors [31], but often corn kernels have been used as substrate since corn is the primary crop infected with *F. verticillioides*.

Recently putative homologues to the *F. verticillioides *fumonisin gene cluster were found in two different *Aspergillus niger *genomes [32,33], and it was subsequently shown that three full genome sequenced strains and the ex type strain of *A. niger *actually can produce FB_2 _in comparable amounts to *Fusarium *strains [34] when grown on agar substrates with high amounts of sugar, glycerol or NaCl.

This was followed by the discovery of additional FB_4 _production (~20% the amount of FB_2_) by *A. niger *[35] in agar cultures and naturally *A. niger *contaminated Thai coffee beans [35].

The objectives of the present work were to *i*) screen *A. niger *and *Fusarium *strains, for production of FB_1_, FB_2 _and FB_3 _on three different agar substrates, *ii*) investigate the effect of incubation temperature on the production of fumonisins and *iii*) study the effect of the solutes glycerol, NaCl and sucrose on the production of fumonisins. The current work is performed on agar media instead of natural substrates in order to more easily asses the water activity.

## Results

### Optimization of extraction

The efficiency of five different extraction solvents to extract FB_2 _from *A. niger *(NRRL 567) varied significantly, with methanol:water (3:1) being most efficient, followed by acetonitrile:water (3:1) with a 20% lower efficiency and methanol:dichloromethane:ethyl acetate (1:2:3) (30% lower efficiency). The use of water (25°C) and hot water (100°C) was not suitable for extraction of FB_2 _from *A. niger *NRRL 567 with a relative efficiency of <1%, when compared to methanol:water. The most effective solvents concerning *F. verticillioides *IBT 9400 were acetonitrile:water (3:1) as the best followed by methanol:water (3:1)(98%) and water at room temperature with an efficiency of 93%. The use of hot water as extraction solvent was less efficient (76%), compared to methanol:water. The acidic methanol:dichloromethane:ethyl acetate (1:2:3), had the lowest extraction efficiency with 26%. The difference between each of the two replicates for all incidences was in the interval 1-40% with an average of 10%.

### Validation of methanol-water extraction from *A. niger*

The recovery of FB_2 _from two spiked non-FB_2 _producing strains showed a recovery of 75% ± 10% (IBT 20381) and 85% ± 10% (IBT 19345). The calibration curves from standards and spiked samples, used to calculate recovery from all had R^2 ^better than 0.995. The relative standard deviation (RSD) of the extracted amounts of FB_2 _from the 8 isolates (n = 5) varied within 4-50%, with an average RSD of 20%. LOD were found to be 0.1 μg/cm^2 ^fungal culture.

### Screening of strains for fumonisin production

The results from the screening experiment are shown in table 1. The *A. niger *strains were able to produce FB_2 _on all three substrates, with the highest production on RC and CYAS. None of the *A. niger *strains produced detectable amounts of neither FB_1 _nor FB_3_. LC-MS/MS analyses have shown that FB_2 _is produced along with FB_4_, although the amount of FB_4 _normally lies in the range 5-20% of the FB_2 _amounts [35].

**Table 1:** Fumonisin production by *Aspergillus niger *and *Fusarium *spp. on CYAS, PDA and RC after 7 days growth at 25°C.

Fungi	Isolate	CYAS μg/ml	RC μg/ml	PDA μg/ml
*A. niger*	NRRL 3	2.9 ± 0.4	7.9 ± 0.7	0.86 ± 0.02

	NRRL 567	25 ± 0.9	36 ± 2	1.9 ± 0.5

	NRRL 2001	7.6 ± 0.7	6.1 ± 0.9	3.1 ± 0.3

	IBT 24631	5.2 ± 0.2	6.7 ± 0.7	1.3 ± 0.6

	IBT 24634	6.4 ± 0.1	5.3 ± 0.4	0.46 ± 0.02

*F. proliferatum*	IBT 8904	n.d.	9.9 ± 3	21 ± 0.5

	IBT 9109	n.d.	0.028 ± 0.007	n.d.

	IBT 9337	n.d.	0.021 ± 0.01	n.d.

	IBT 9393	n.d.	0.03 ± 0.001	2.0 ± 0.04

	IBT 9397	n.d.	46 ± 3	33 ± 0.5

	IBT 41107	n.d.	5.4 ± 1	6.7 ± 0.6

*F. verticillioides*	IBT 9400	n.d.	0.035 ± 0.005	35 ± 0.7

	IBT 9492	n.d.	0.028 ± 0.004	4.9 ± 0.2

	IBT 9496	n.d.	0.033 ± 0.0003	n.d.

	IBT 9502	n.d.	2.2 ± 0.4	18 ± 1

	IBT 9505	n.d.	0.078 ± 0.09	9.5 ± 3

	IBT 41110	n.d.	0.12 ± 0.07	4.5 ± 0.7

*F. dlaminii*	IBT 2937	n.d.	n.d.	n.d.

	IBT 2938	n.d.	n.d.	n.d.

*F. napiforme*	IBT 2931	n.d.	0.24 ± 0.06	6.2 ± 0.9

	IBT 2932	n.d.	0.13 ± 0.1	0.081 ± 0.02

*F. nygamai*	IBT 2933	n.d.	0.033 ± 0.006	n.d.

	IBT 2934	n.d.	22 ± 2	5.6 ± 0.6

	IBT 8290	n.d.	0.041 ± 0.006	0.047 ± 0.001

	IBT 8554	n.d.	0.033 ± 0.01	0.039 ± 0.003

	IBT 8557	n.d.	0.01 ± 0.01	0.14 ± 0.002

	IBT 8566	n.d.	3.1 ± 1	6.2 ± 0.5

	IBT 9394	n.d.	0.30 ± 0.08	n.d.

	IBT 9395	n.d.	0.03 ± 0.003	16 ± 0.6

*F. oxysporum*	IBT 9514	n.d.	2.8 ± 0.4	37 ± 0.8

The concentration of FB_2 _was detected in the methanol:water (3:1) extract. The values are means of the replicates plus/minus the standard deviation. The replicates are made in biological duplicates on two separate plates.

n.d. not detected.

Standard deviation calculated on two measurements

Of the *A. niger *strains, NRRL 567 had the highest production of FB_2 _on RC and CYAS. But on PDA the three strains, NRRL 567, NRRL 2001 and IBT 24631, produced very similar amounts of fumonisins, although lower than RC and CYAS. The FB_2 _production on RC and CYAS of *A. niger *NRRL 2001, IBT 24631 and 24634 differed only slightly, whereas FB_2 _production by the other two, NRRL 567 and NRRL 3 were clearly favored by growth on RC. All *Fusarium *spp. with the exception of the two *F. dlaminii *strains IBT 2937 and IBT 2938 produced fumonisins under these conditions. Six *Fusarium *strains, *F. napiforme *IBT 2932, *F. proliferatum *IBT 9109 and IBT 9337, *F. verticillioides *IBT 9496 and *F. nygamai *IBT 9395 produced amounts close to the detection limit of FB_1 _on RC; in addition IBT 2932 also produced fumonisins on PDA. *F. nygamai *IBT 2934, IBT 8554 and IBT 8557 showed a higher production of FB_2 _than FB_1 _on PDA (data not shown), and *F. proliferatum *IBT 9397 had the highest concentration of total fumonisin measured. Only seven strains had a total production of fumonisin above 1 μg/ml extract on RC compared to 14 strains on PDA.

Seven *Fusarium *strains did not have any measurable production of FB_1_, FB_2 _nor FB_3 _on PDA compared to two non-producers on RC. On the other hand PDA supported production of higher amounts of fumonisins, strains which had a barely detectable amount of FB_1 _on RC, also showed production of either FB_2 _or both FB_2 _and FB_3 _on PDA.

Strains for the next experiments were selected on the basis of the above mentioned experiments. Besides the five *A. niger *strains five *Fusarium *strains were selected, with both good and poor producers at 25°C, two strains with a high FB production, *F. verticillioides *IBT 9400 and *F. oxysporum *IBT 9514, one with an average FB production *F. proliferatum *IBT 41107, one with a low FB_1 _production *F. napiforme *IBT 2932 and a strain with a higher production of FB_2 _than FB_1 _*F. nygamai *IBT 8554 was selected.

### The effect of temperature on growth and production of fumonisins by *A. niger *and *Fusarium *spp

**Figure 1:**
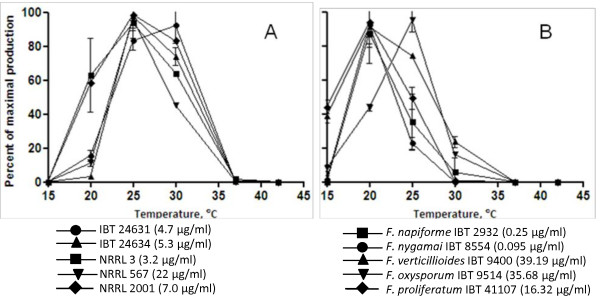
**A: FB_2 _production by *Aspergillus niger *after 7 days of growth at different temperatures (15-42°C) on CYAS**. The concentration of FB_2 _was detected in the methanol:water (3:1) extract. The values are means of biological duplicates on two different plates; highest value (μg/ml) is in parenthesis. B: Total production of fumonisin B_1_, B_2 _and B_3 _produced by *Fusarium *spp. after 7 days growth at different temperatures (15-42°C) on PDA. The concentration of fumonisin was detected in the methanol:water (3:1) extract. The values are means of biological duplicates on two different plates; highest value (μg/ml) is in parenthesis.

Only one of the *A. niger *strains was able to grow at 15°C, although very slowly (extensive data shown in [Additional file 1]). The growth increased at higher temperature and peaked at 30-37°C, followed by a slight reduction of the growth at 42°C. All *Fusarium *strains were able to grow at 15°C followed by an increased growth at higher temperatures and peaked at 25-30°C, above this temperature the growth decreased and no growth was observed at 42°C. The effect of temperature on the production of FB_2 _by the five *A. niger *strains is shown in figure 1A. None of the isolates produced detectable amounts of FB_2 _at 42°C, even though all strains grew well. The only *A. niger *strain, NRRL 2001, that was able to grow at 15°C, did not have any detectable production of FB_2_.

At 37°C, the optimal temperature for growth by *A. niger*, the FB_2 _production was very limited. NRRL 567 had the highest production of the five strains at 25°C and 30°C; however at 20°C NRRL 2001 showed the highest production. This strain showed also the highest growth rate at this temperature. Four strains, NRRL 567, NRRL 3, NRRL 2001 and IBT 24634 had the highest FB_2 _production at 25°C, followed by 30°C, 20°C, and 37°C (Figure 1A). One strain, IBT 24631, differed from the other four, since it had a maximal production at 30°C, followed by 25°C, 20°C and 37°C.

The concentration of FB_1_, FB_2 _and FB_3 _produced by *Fusarium *spp. is shown in figure 1B, and four of the *Fusarium *spp. had maximal production at 20°C: *F. verticillioides *IBT 9400, *F. proliferatum *IBT 41107, *F. napiforme *IBT 2932 and *F. nygamai *IBT 8554. *F. oxysporum *IBT 9514 had a maximal production of fumonisin at 25°C. Only three of the *Fusarium *strains, *F. verticillioides *IBT 9400, *F. oxysporum *IBT 9514 and *F. proliferatum *IBT 41107 had a measurable production of fumonisin at 15°C. *F. napiforme *IBT 2932 was only able to produce detectable amounts of fumonisins in the temperature range 20-25°C. At 20°C this strain had detectable concentrations of FB_1_, FB_2 _and FB_3_, but at 25°C only FB_1 _and FB_2 _were detected (data not shown). Even though there was growth of all five *Fusarium *strains at 37°C there were only in one case detectable production of fumonisins, this was produced by *F. verticillioides*.

### The effect of glycerol, NaCl and sucrose on the growth and production of fumonisins by *A. niger *and *Fusarium *spp

**Figure 2:**
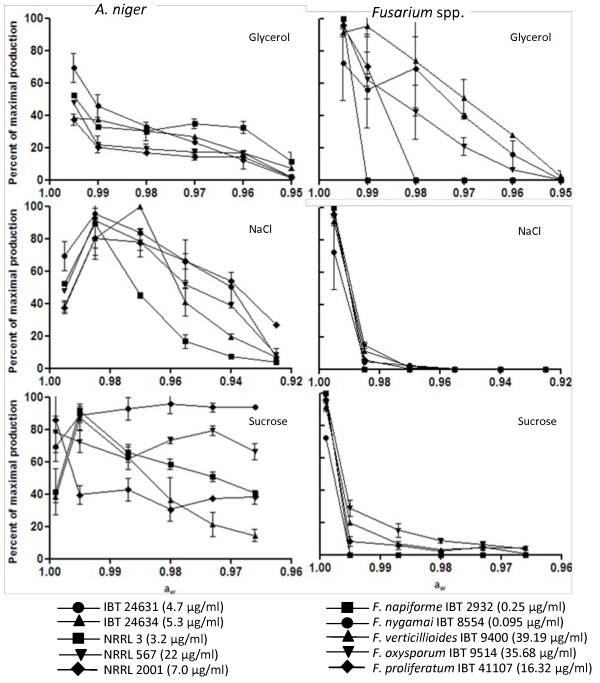
**Effect of glycerol, NaCl and sucrose on the FB_2 _production of *Aspergillus niger *and the total production of FB_1_, FB_2 _and FB_3 _by *F. verticillioides, F. proliferatum*, *F. napiforme*, *F. nygamai *and *F. oxysporum***. Strains have been incubated at 25°C for 7 days on CYA or PDA. The concentration of fumonisins were detected in the methanol:water (3:1) extract. The values are means of biological duplicates on two different plates, highest value is in parenthesis.

All strains of *A. niger *and *Fusarium *spp. were able to grow at all glycerol concentration (0-255 g/l) [see Additional file 2]. The growth of *A. niger *was only slightly reduced at a_w _0.99; below this there was a continuous decrease in the growth. For the *Fusarium *spp. the growth was reduced at a_w _0.99 and below, which was the same as the *A. niger*. All *A. niger *strains were able to produce FB_2 _at all glycerol concentrations tested (Figure 2). The effect of glycerol on the quantitative FB_2 _production for *A. niger *was very strain dependent, but in general the average FB_2 _production was reduced 8.2% per 0.01 a_w _unit (R^2 ^= 0.97). Three of the five *Fusarium *strains were able to produce fumonisin at all glycerol concentrations: *F. nygamai *IBT 8554, *F. oxysporum *IBT 9514 and *F. verticillioides *IBT 9400 (Figure 2). For two of these, IBT 8554 and IBT 9400, fumonisin production was increased up to 20% when glycerol was added. The other two strains *F. napiforme *IBT 2932 and *F. nygamai *IBT 8554 did not have a measurable production of fumonisins at a_w _0.99 and 0.98. The average total fumonisin production was reduced 18% per 0.01 a_w _unit (linear regression R^2 ^= 0.91).

For the five *A. niger *growth in the presence of NaCl was partially inhibited below a_w _0.98 and all strains were able to grow at all a_w _tested. The *Fusarium *species had the highest growth rates at the highest a_w _and the growth declined until a_w _0.93 where they were unable to grow.

The FB_2 _production of all *A. niger *strains was stimulated when NaCl was added with FB_2 _being detected at all NaCl concentrations (Figure 2). The production peaked at a_w _0.985-0.97 depending on the strain. A reduction of the FB_2 _production started at a_w _0.97-0.94 and a comparison by a regression analysis to the cultures with no NaCl added the average production of FB_2 _decreased 13% per 0.01 a_w _unit (R^2 ^= 0.98). The addition of NaCl clearly reduces the production of fumonisins by *Fusarium *(Figure 2), and was not detected in any culture below a_w _0.955. One strain, *F. napiforme *IBT 2932, did not even have a measurable production of fumonisin when NaCl was added. But no correlations in the average decrease of fumonisin production was observed.

The *A. niger *and *Fusarium *spp. strains grew better on sucrose, compared to NaCl and glycerol. The addition of sucrose increased the production of FB_2 _for all the *A. niger *strains (Figure 2). The increase in the FB_2 _production was followed by either a reduction or stagnation in the production level at higher sucrose concentrations. The average decrease in the total fumonisin production were 8.3% per 0.01 a_w _unit (R^2 ^= 0.87)

Only three of the five *Fusarium *strains were able to produce FB_1_, FB_2 _and FB_3 _when cultured on different sucrose concentrations (Figure 2). Because a commercial potato extract without sucrose was not obtainable, the base potato extract was prepared on home-made boiled potatoes. The three *Fusarium *strains with a detectable fumonisin production were *F. oxysporum *IBT 9514, *F. proliferatum *IBT 41107 and *F. verticillioides *IBT 9400. *Fusarium oxysporum *IBT 9514 had a maximal production at a_w _0.995, while *F. proliferatum *IBT 41107 peaked at a_w _0.999 and *F. verticillioides *IBT 9400 peaked at a_w _0.999. A reduction of the fumonisin production was observed at higher sucrose concentrations. There were no correlations in the average decrease of fumonisin production

## Discussion

We found that *A. niger *was able to produce FB_2_, in agreement with Frisvad *et al*. [34] who showed that *A. niger *produced FB_2 _on the agar substrates RC and CYAS. On the other hand they did not measure any production of FB_2 _on PDA, whereas all five *A. niger *strains in the present study had a detectable, albeit low, production of FB_2 _on this substrate. Besides *F. verticillioides*, also *F. napiforme*, *F. nygamai*, *F. proliferatum *and *F. oxysporum *were found to produce fumonisins on laboratory agar substrates. The ability to produce fumonisins by these species correlates with findings of Nelson *et al*. [8] and Kpodo *et al*. [9]. However Nelson *et al*. [8] described the production of FB_1 _by *F. dlaminii*, but this was not supported in our study. The findings of strains, capable of producing more FB_2 _than FB_1 _was also described by Musser & Plattner [36] as well as Leslie *et al*. [37]. Apart from the four *A. niger *strains shown to produce FB_2 _by Frisvad *et al*. [34], additional 12 strains did produce this mycotoxin. Among the 18 *A. niger *strains investigated until now, only two have been unable to produce fumonisins in detectable amounts on the media investigated.

Astoreca *et al*. [38] found the optimal temperature for growth of *A. niger *to be 30°C, the highest investigated temperature in their study. In correlation to this study Palacios-Cabrera *et al*. [39] also found that *A. niger *grew optimally at temperatures of 30°C, which was also the optimal temperature for linear growth in our study. Marin *et al*. [40] found that the growth of both *F. verticillioides *and *F. proliferatum *was best at 25 to 30°C, which is in agreement of our results. According to Marin *et al*. [40]*F. verticillioides *is more tolerant to temperature above 30°C than other *Fusarium *spp., however this was not obvious in our study.

We found that the optimal production of FB_2 _by *A. niger *was at 25°C and in one case 30°C. Since there has been only one report of the fumonisin production by *A. niger *we have compared our results to the production of ochratoxin A. Our results correlates with an investigation of Esteban *et al*. [41], who showed that the optimal temperature for production of ochratoxin A by *A. niger *was at 20-25°C. In contrast to this, other authors found that the optimal ochratoxin A production in a synthetic grape juice medium was significantly better at 15°C compared to both 25 and 35°C [42]. Findings in our study showed that at 20°C a significant decrease in the FB_2 _production occurred compared to 30°C. Earlier studies show that the optimal temperature for production of fumonisins by *F. proliferatum *is at 15-20°C where *F. verticillioides *prefers the higher temperatures of 30°C [27]. This partly correlates with the results from our study, where both isolates showed the highest production at 20°C, but also produced fumonisins at 30°C. However fumonisin production by *F. verticillioides *was less inhibited than *F. proliferatum *by the higher temperature. Dilkin *et al*. [26] and Alberts *et al*. [25] found the optimal temperature for fumonisin production to be 25°C, followed by 20 and then 30°C. These results deviate from our results, because four isolates had the best production at 20°C and one at 25°C. Marin *et al*. [27] described the production of FB_1 _by *F. verticillioides *at 37°C, which was also observed in our study. From our results a general pattern in the fumonisin production for both genera was observed, namely the maximal production of fumonisins being 5°C below the optimal growth.

Comparing *A. niger *with the *Fusarium *spp., all *A. niger *strains grew better at all tested a_W _values. Leong *et al*. [42] found the optimal a_w _for ochratoxin A production by *A. niger *to be a_w _0.95, whereas Esteban *et al*[43] found it to be in the range of 0.96-0.99, and that it was very strain dependent. These values are lower than those observed in our study for fumonisins where four of the *A. niger *strains had the highest production of FB_2 _at a_W _0.99, with one isolate produced most FB_2 _at a_w _0.98. Earlier studies have shown that the optimal a_W _for fumonisin production by *Fusarium *is in the interval 0.97-0.98 [27,44]. The optimal a_w _value from our study was a_W _0.995, which is a bit higher than the above mentioned a_w _values. Frisvad *et al*. [34] also found that the addition of 5% NaCl (a_w _= 0.97) or 20% sucrose (a_w _= 0.99) increased the production of FB_2 _by *A. niger*. The present study showed that not all the used strains had the same pattern. Only four of the five strains had an increase in FB_2 _production at a_w _0.97 compared to the zero sample when grown on NaCl. The last *A.niger *NRRL 3 had only an increase at a_w _0.985 followed by a decrease at higher NaCl concentrations. The same was observed with sucrose where three strains had an increase in the FB_2 _production compared to a sucrose concentration of 3% in standard medium. The last strains had a decrease in the production at the previous mentioned sucrose concentrations. In conclusion it is clear that there is very large strain variability in fumonisin production at different water activities between the *A. niger *strains used in these experiments.

Further studies on the effect of physiological variables on fumonisin production by *A. niger *is needed on large numbers of strains, due to large strain differences, and should further be backed up by studies of commodities where *A. niger *is common. This will lead us to a better understanding of how large a food safety problem fumonisin production by *A. niger *is. Only one report of fumonisins from *A. niger *in food (green coffee beans) has been reported, however the amounts were well below the regulatory limit [35]. Fumonisin contamination in a series of foods, often contaminated with *A. niger*, may show to be an overlooked mycotoxin problem, since surveys have only mostly targeted corn and rice, based on the belief that it was only *Fusarium *spp. that could produce these important mycotoxins.

## Conclusion

The present study shows that the regulation of fumonisin production is very different in *A. niger *compared to *Fusarium*, with the latter preferring high a_w _(>0.99) and low temperature (20-25°C) and with *A. niger *preferring lower a_w _and higher temperature (25-30°C). Fumonisin produced by *A. niger *may be an overlooked health risk in foods not previously associated with fumonisins, especially because *A. niger *is known as a common food spoilage organism on a wide variety of foods [45].

## Methods

Unless otherwise is stated all solvents were HPLC grade, chemicals were analytical grade and water was purified on a Milli-Q system (Millipore, Bedford, MA). Media were prepared in 9 cm Petri dishes, each with 20 ml medium, and strains were inoculated by single point and dishes incubated in micro perforated plastic bags at 25°C for 7 days in darkness. The colony diameter measured is an average of the smallest and broadest diameter. All samples were as a minimum made in biological duplicates on two individually plates.

### Fungal strains and media

All strains (Table 2) were from the IBT culture collection at Center for Microbial Biotechnology, DTU. The *Fusarium *strains were selected from species known to produce fumonisin. Five *A. niger *strains were selected, for the physiologically study, three of these have been used in industry according to collection databases (NRRL 3, NRRL 567 and NRRL 2001), whereas the last two were isolated from black pepper (IBT 24634, IBT 24631). The last 9 strains were only used for validation. All *A. niger *strains were characterized and identified using a polyphasic approach, as in our previous articles [46,47], in order to ensure that they were *A. niger sensu stricto*. Besides this the ITS region and parts of the β-tubulin and calmodulin genes were amplified and sequenced as described previously [48-50]. Results shown in Table 2 (public database numbers of the strains).

**Table 2:** Fungal isolates used for fumonisin production.

Fungi	Isolate	Genbank numbers
*Aspergillus niger*	NRRL 3 (ex unknown) (= ATCC 9069, CBS 120.49, IBT 23539) (Full genome sequenced)	FJ639289
	NRRL 567 (ex unknown) (= ATCC 12846, IBT 26387)	GU195638
	NRRL 2001 (ex unknown) (= ATCC 13794, IBT 26392)	GU195639
	IBT 24631(ex black pepper)	GU195636
	IBT 24634 (ex black pepper)	GU195637

Only used for validation	IBT 4983 (ex unknown) (= CBS 117.80)	GU195632
	IBT 18741 (ex carpet dust)	FJ639294
	IBT 19345 (ex unknown) (= IFO 6082)	GU195633
	IBT 19558(ex coffee beans)	GU195634
	IBT 20381 (ex coffee beans)	GU195635
	IBT 26774 (ex unknown)	
	IBT 28086 (ex grape)	
	IBT 28104 (ex black pepper)	GU105640

*Fusarium*	IBT 2937 (ex plant debris) (= FRC M-1688)	
*dlaminii*	IBT 2938 (ex plant debris) (= FRC M-1638)	

*F. napiforme*	IBT 2931 (ex soil debris from grassland) (= FRC M-1647)	
	IBT 2932 (ex soil debris from grassland) (= FRC M-1646)	

*F. nygamai*	IBT 2933 (ex unknown) (= FRC M-2376)	
	IBT 2934 (ex root debris from grassland) (= FRC M-2371)	
	IBT 8290 (ex unknown) (= MRC 4373)	
	IBT 8554 (ex unknown)	
	IBT 8557 (ex unknown)	
	IBT 8566 (ex corn kernel)	
	IBT 9394 (ex unknown) (= MRC 3997)	
	IBT 9395 (ex unknown) (= MRC 3998)	

*F. oxysporum*	IBT 9514 (ex corn kernel)	

*F. proliferatum*	IBT 8904 (ex yellow onion)	
	IBT 9109 (ex barley)	
	IBT 9337(ex corn stalk)	
	IBT 9393 (ex unknown) (= MRC 3218)	
	IBT 9397 (ex unknown) (= MRC 3216)	
	IBT 41107 (ex corn)	

*F. verticillioides*	IBT 9400 (ex unknown) (= MRC 826)	
	IBT 9492 (ex corn kernel)	
	IBT 9496 (ex corn kernel)	
	IBT 9502 (ex corn kernel)	
	IBT 9505 (ex corn kernel)	
	IBT 41110 (ex corn)	

ATCC: American Type Culture Collection, Manassas, VA, USA

CBS: Centraalbureau voor Schimmelcultures, Utrecht, The Netherlands.

FRC: Fusarium Research Center, Penn State University, University Park, Pennsylvania, USA

IFO: Institute for Fermentation, Osaka, Japan.

MRC: South African Medical Research Council, Tygerberg, South Africa.

NRRL: Northern Regional Research Laboratory, Peoria, Illinois, USA.

The media used for fumonisin production were: potato dextrose agar (PDA) [51], Czapek yeast autolysate agar with 5% NaCl (CYAS) [52] and rice meal corn steep liquor (RC) agar [53].

### Fumonisin analysis

The fumonisin were extracted using the method previously described by Frisvad *et al*. [34]. Six plugs (D = 6 mm) were cut out of the colony from the center and in a radius towards the edge of the colony and transferred to a clean 2-ml vial, 800 μl of methanol:water (3:1) was added, and extracted by ultrasonication for one hour. All extracts were filtered through a 13 mm PTFE 0.45 μm syringe filter (National Scientific, Rockwood, Tennessee) into a new vial and used directly for analysis.

The LC-MS analysis was performed on a LC/MSD VL single quadrupole (Agilent, Santa Clara, California). The separation of 3 μl extracts were done at 40°C on a 50 × 2 mm, i.d. 3 μm size, Luna C-18 (II) column (Phenomenex, Torrance, California), fitted with a security guard column, and using a water:acetonitrile (both containing 20 mM formic acid) gradient at a flow rate of 0.3 ml/min. The gradient started at 30% acetonitrile, and increased to 60% acetonitrile over 5 minutes. During further 1 minute it was increased to 100% acetonitrile and maintained here for 2 minutes before the gradient in 1 minute was returned to starting conditions and kept there for 5 minutes. The mass spectrometer (MS) was operated in positive electrospray ionization mode and was automatically calibrated on the instrument ESI tuning mix. The MS was used in selected ion monitoring (SIM) mode for measuring [M+H]^+^: FB_1 _(m/z 722), FB_2 _and FB_3 _(m/z 706). The capillary voltage was held at 3000 V, the fragmentor voltage was at 70 V and the nebulizer pressure was at 2.5 bar. The drying gas flow was 12 l/min with a temperature of 350°C. The detection limit was measured to 0.01 μg/ml from dilutions of a FB_1 _and FB_2 _certified standard (Biopure, Tulin, Austria), with concentrations of 50.2 μg/ml and 51.0 μg/ml, respectively. The fumonisin concentrations of the extracts were calculated from a standard curve created from dilutions of the FB_1 _and FB_2 _standard mixture. FB_2 _presence was further confirmed in selected extracts by LC-MS/MS [35] and LC-HRMS [34].

### Efficiency of extraction solvents

The five different extraction solvents tested on *A. niger *NRRL 567 and *F. verticillioides *IBT 9400 were: *i*) methanol:water (3:1), *ii*) acetonitrile:water (3:1), *iii*) water at room temperature (25°C), *iv*) water at 100°C and *v*) methanol:dichloromethane:ethyl acetate (1:2:3) with addition of 1% (v/v) formic acid. The extraction process for [*i-iv*] was the same as mentioned above. For the fifth extraction solvent there were a few extra steps: After ultrasonication the extract was transferred to a new vial and the organic phase was evaporated *in vacuo*. The residue was re-dissolved by ultrasonication in 500 μl methanol for 20 minutes. All extracts were filtered through a PTFE 0.45 μm syringe filter before analysis.

### Validation of methanol:water extraction

FB_2 _extraction was validated by spiking 5 plugs of two non-fumonisin producing strains of *Aspergillus niger *(IBT 19345 and IBT 20381) with 100 μl FB_2 _standard containing 5000, 2500, 1000, 500 and 100 ng FB_2_. After spiking, the plugs were left for 2 hours, and extracted as described above. Recovery was determined by comparing the slope of the spiked curve to slope of the curve from diluted samples in methanol-water (3:1). Five blank samples of the 2 strains were also analysed.

Reproducibility of extraction efficiency was further determined from 7 *A. niger *strains (IBT 4983, IBT 18741, IBT 19558, IBT 26774, IBT 28086, IBT 28104, NRRL 567) selected from high, medium and low fumonisin producing strains. Five replicate plates were made from each strain and extracted as described above.

### The effect of temperature on the growth and production of fumonisin by *A. niger *and *Fusarium *spp

To assess the effect of temperature on the production of fumonisins, *A. niger *strains were inoculated on CYAS and *Fusarium *strains were inoculated on PDA. The plates were incubated in darkness at 15, 20, 25, 30, 37 or 42°C respectively for 7 days. The fungi used were the five *A. niger *strains listed in Table 2, and the following *Fusarium *species: *F. napiforme *IBT 2932, *F. nygamai *IBT 8554, *F. verticillioides *IBT 9400, *F. oxysporum *IBT 9514 and *F. proliferatum *IBT 41107.

### The effect of glycerol, NaCl and sucrose on the growth and production of fumonisin by A. niger and Fusarium spp

For investigation of the effect of glycerol, NaCl and sucrose on the production of fumonisin the following experiments were performed: *A. niger *was inoculated on CYA and *Fusarium *on PDA with different concentration of glycerol (0-255 g/l), salt (0-125 g/l) and sucrose (0-530 g/l). For the *Fusarium*-sucrose experiment, the PDA mixture was made from boiled potatoes instead of a commercial blend. In short terms, 200 g potatoes was peeled and diced and autoclaved at 121°C with 1 liter of water, 15 g agar and 0-530 g sucrose.

The corresponding measured water activity (a_W_) values of the media are shown in Table 3. The water activity was measured with an Aqualab (ADAB Analytical Devices, Stockholm, Sweden). There were no measurable differences in the water activity of the CYA and PDA media. The fungi used were five *A. niger *strains, and the following *F. napiforme *IBT 2932, *F. nygamai *IBT 8554, *F. verticillioides *IBT 9400, *F. oxysporum *IBT 9514 and *F. proliferatum *IBT 41107.

**Table 3:** The concentration of glycerol, NaCl and sucrose and the corresponding measured water activity (a_w_)

Glycerol (g/l)	0	51	102	154	204	255	---
a_w_	1 ± 0	0.99 ± 0.0015	0.98 ± 0	0.97 ± 0.001	0.96 ± 0.001	0.95 ± 0.001	---

NaCl (g/l)	0	25	50	75	100	125	---

a_w_	1 ± 0	0.985 ± 0.0006	0.97 ± 0.001	0.955 ± 0.0006	0.94 ± 0.0006	0.92 ± 0.0015	---

Sucrose (g/l)	0	30	130	230	330	430	530

a_w_	1 ± 0	0.999 ± 0.0006	0.995 ± 0.0015	0.987 ± 0.0015	0.98 ± 0	0.973 ± 0.001	0.967 ± 0.001

The values are means of the triplicates plus/minus the standard deviation.

## Authors' contributions

JMM, UT and JCF designed the study. JMM performed the experiments, KFN the analytical part and RAS the molecular genetic studies. All authors contributed in drafting the paper and have read and approved the final manuscript.

## Supplementary Material

Additional file 1 **Effect of temperature on the growth of *Aspergillus niger *and *Fusarium *spp**. The conidial diameter of 5 *Fusarium *spp. and 5 *Aspergillus niger *strains at different temperature in the range of 15-42°C after 7 days growth. Click here for file

Additional file 2 **Effect of water activity on the growth of *Aspergillus niger *and *Fusarium *spp**. The conidial diameter of 5 *Fusarium *spp. and 5 *Aspergillus niger *strains at different a_w _in the range of 0.92-1 after 7 days growth. Click here for file
