# Supplementary material for: Effect of temperature and water activity on the production of fumonisins by Aspergillus niger and different Fusarium species
Source: BMC Microbiol. 2009 Dec 31;9:281. doi: 10.1186/1471-2180-9-281 (PMC2811119; doi:10.1186/1471-2180-9-281)
Supplement: Additional file 1 — Effect of temperature on the growth of Aspergillus niger and Fusarium spp. The conidial diameter of 5 Fusarium spp. and 5 Aspergillus niger strains at different temperature in the range of 15-42°C after 7 days growth. [file 1471-2180-9-281-S1.DOCX]

# Additional file 1

The conidial diameter of 5 *Fusarium* spp. and 5 *Aspergillus niger* isolates at different temperature in the range of 15-42 °C. All isolates were grown in perforated plastic bags at 25°C for 7 days in darkness.

The diameter is an average of the smallest and broadest conidial diameter.

*F. napiforme* 2932
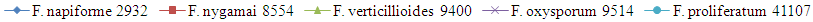
*F. nygamai* 8554
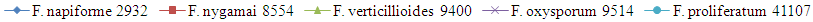
 *F. verticillioides* 9400
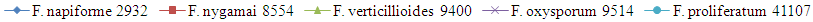
 *F. oxysporum* 9514


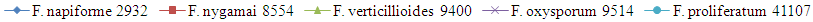


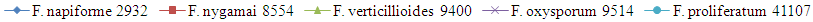
 *F. proliferatum* 41107

*A. niger* :
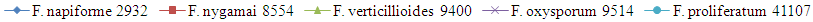
 NRRL 3
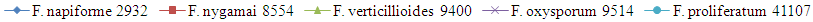
NRRL 567
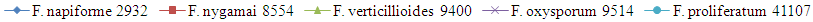
 NRRL 2001
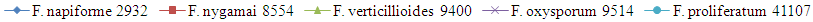
 IBT 24631
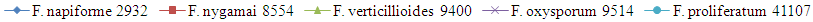
 IBT 24634
